# High-quality-factor dual-band Fano resonances induced by dual bound states in the continuum using a planar nanohole slab

**DOI:** 10.1186/s11671-021-03607-x

**Published:** 2021-09-28

**Authors:** Qing Mi, Tian Sang, Yao Pei, Chaoyu Yang, Shi Li, Yueke Wang, Bin Ma

**Affiliations:** 1 Department of Photoelectric Information Science and Engineering, School of Science, Jiangnan University, Wuxi, 214122 China; 2 Key Laboratory of Advanced Micro-Structured Materials MOE, Institute of Precision Optical Engineering, School of Physics Science and Engineering, Tongji University, Shanghai, 200092 China

**Keywords:** Fano resonance, Bound states in the continuum, High quality factor, Tetramerized holes, Planar nanohole slab

## Abstract

In photonics, it is essential to achieve high-quality (Q)-factor resonances to improve optical devices’ performances. Herein, we demonstrate that high-Q-factor dual-band Fano resonances can be achieved by using a planar nanohole slab (PNS) based on the excitation of dual bound states in the continuum (BICs). By shrinking or expanding the tetramerized holes of the superlattice of the PNS, two symmetry-protected BICs can be induced to dual-band Fano resonances and their locations as well as their Q-factors can be flexibly tuned. Physical mechanisms for the dual-band Fano resonances can be interpreted as the resonant couplings between the electric toroidal dipoles or the magnetic toroidal dipoles based on the far-field multiple decompositions and the near-field distributions of the superlattice. The dual-band Fano resonances of the PNS possess polarization-independent feature, and they can be survived even when the geometric parameters of the PNS are significantly altered, making them more suitable for potential applications.

**Supplementary Information:**

The online version contains supplementary material available at 10.1186/s11671-021-03607-x.

## Introduction

Enhancing the interaction between light and matter, which is significant for improving performances of optical devices, can be realized by using high-quality (Q)-factor responses [1]. Fano resonance, characterized by the asymmetric line shape and sharp spectral profile, provides an effective approach to achieve the high Q-factor in optical metamaterials and has received great attention [2]. In the last decade, Fano resonance has been reported in many nanoscale oscillator systems enabled by plasmonic nanostructures [3, 4], where Fano resonance is excited by the surface plasma resonance at the metal–dielectric interface. Although metallic metamaterials are promising candidates for light manipulation, Fano resonance in plasmonic metamaterials typically suffers from low Q-factor in the visible to near-infrared (NIR) spectral regions due to the inherent ohmic losses in metal.

On the other hand, all-dielectric metamaterials provide strong Mie-type resonances with induced displacement currents similar to those of plasmonic metamaterials, but feature less dissipative losses in the visible to NIR range [5]. The energy of the incident light can be highly localized in the dielectric nanostructures due to the excitation of the electric and/or magnetic dipolar resonances, which reduces the dissipative losses and achieves large resonant enhancement of both electric and magnetic fields. In recent years, bound states in the continuum (BICs) have emerged as the most promising scheme for achieving high-Q-factor responses in all-dielectric metamaterials [6, 7]. BICs reside inside the continuous spectrum of extended states but counterintuitively remain perfectly localized in space with theoretically infinite lifetime [8, 9]. Although BICs are not observable from the continuous spectrum due to the non-radiative property, high-Q-factor Fano resonances can be achieved as BICs are transformed into quasi-BICs (QBICs) [10, 11], potential applications include such as directional lasing [12], optical filters [13], nonlinear frequency conversion [14], ultra-sensitive sensors [15, 16] and optical vortex beams [17].

Generally, the formation of BICs is strongly related to the symmetries (in plane and vertical symmetry) of the photonic structure due to its interferential nature. More specially, BICs can be perturbed via oblique incidence or symmetry-broken nanostructures, and the QBICs can be realized as the radiation channel between the eigenstates and the free space is opened [18, 19]. However, most of the dielectric nanostructures used to excite QBICs with high Q-factor are complicated, such as asymmetrical nanocrosses [20], asymmetrical nanorings [21], asymmetrical nanobars [22–24] and asymmetrical nanorods [25–28], which are challenging in fabrication due to the requirement of inserting the deep subwavelength slits [20–24] or nanoholes [25–28] into the photonic structures. Other nanostructures such as the reshaped rectangular bars [29, 30] have the increased sharp edges, making them more difficult to be accurately fabricated through conventional lithographic techniques, which reduces the Q-factor and the resonance lifetime of the devices due to the opening of additional leaky channels [31, 32]. Moreover, the tilted nanobars [33, 34], another type of structures, have difficulties in precisely control of the orientation of the nanobars with the deep subwavelength spaces between the resonators maintained in nanofabrication process. In applications, it is meaningful to realize BICs and high-Q-factor Fano resonances using all-dielectric metamaterials with simpler architectures such as the nanostructured planar slabs [35–38]. Besides, multiple Fano resonances are very useful in the applications such as enhancing multiband harmonic generation [39], multichannel sensing [40] and light emission [41]. Therefore, there is a significant benefit to achieve high-Q-factor multiple Fano resonances using a comparatively simple architecture based on the excitation of QBICs.

In this work, a novel planar nanohole slab (PNS) consisting of tetramerized holes is proposed to achieve high-Q-factor dual-band Fano resonances. By shrinking or expanding the tetramerized holes of the PNS along the diagonals of the superlattice, two QBICs are excited and the locations of the two Fano resonances as well as their Q-factors can be flexibly tuned. Far-field multiple decompositions and near-field distributions of the superlattice are performed to reveal the resonant features of the PNS, indicating that the dual-band Fano resonances are resulted from the resonant couplings between the electric toroidal dipoles or the magnetic toroidal dipoles. The dual-band Fano resonances of the PNS possess polarization-independent feature, and they can be survived even the geometric parameters of the PNS are significantly altered, making it more suitable for potential applications.

## Methods

### Lattice structure and design

**Fig. 1:**
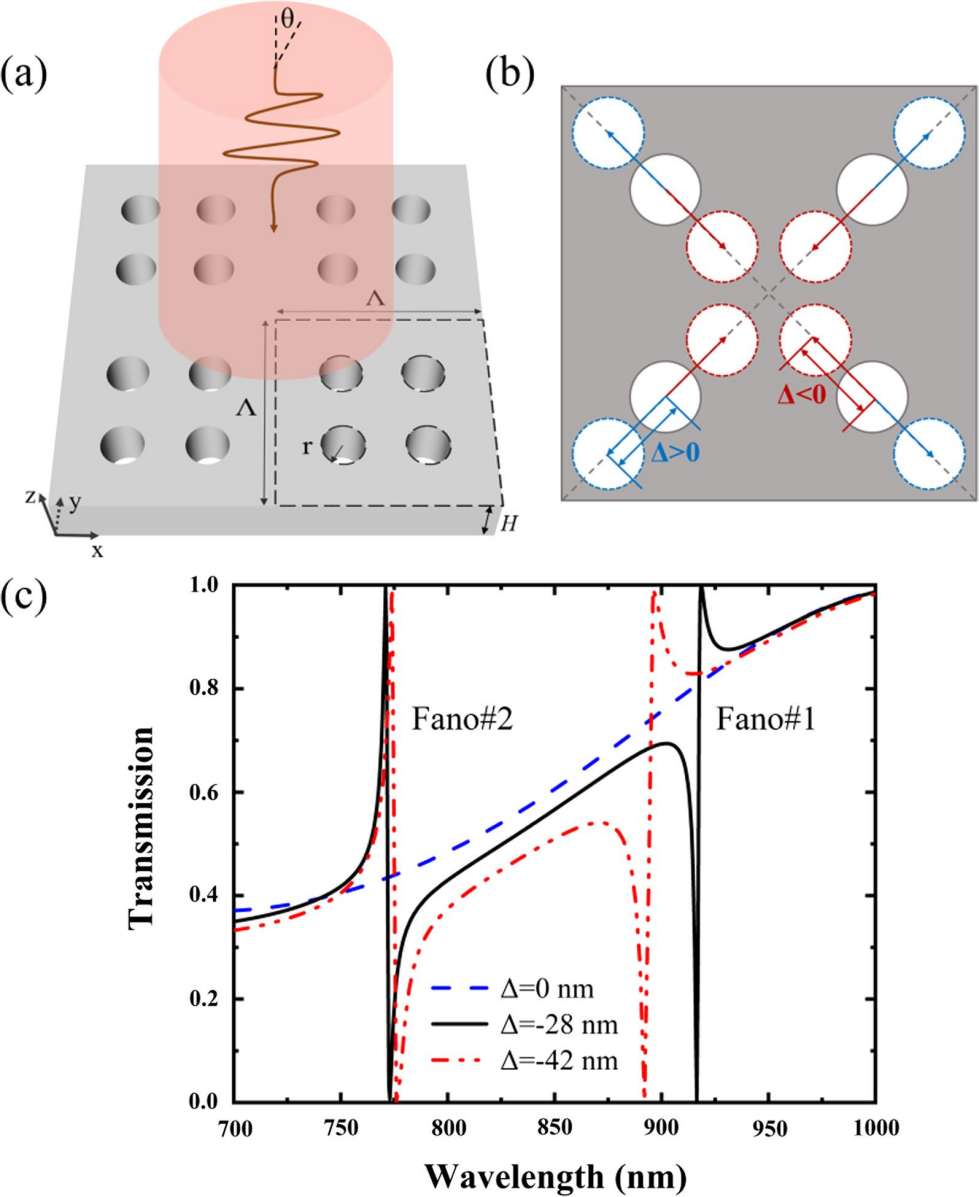
**a** Perspective view of the PNS. **b** Vertical view of the PNS as four nanoholes shrink (Δ < 0) or expand (Δ > 0) along the diagonals of the superlattice. **c** Transmission spectra of the PNS as a function of the shift distance of Δ. The PNS is under the illumination of the *x*-polarized incident wave with the incident angle of *θ* = 0. The parameters of the PNS are: Λ = 350 nm, *r* = 35 nm and H = 175 nm

Figure 1 shows the schematic geometry of the proposed PNS and its transmission spectra. The PNS consists of four nanoholes which can be shrunk (Δ < 0) or expanded (Δ > 0) with a shift distance of Δ along the diagonals of the superlattice, and Δ = 0 corresponds to simple lattice with period reduced to half, where each nanohole is located in the center of a quarter area of the superlattice. The period and the height of the PNS are Λ and H, respectively; the radius of the nanohole is *r*. The refractive index of the PNS is *n*_*s*_ = 3.2, and the background is air with the refractive index of *n*_*a*_ = 1. Figure 1c shows the spectra of the PNS as a function of the shift distance of Δ, where the PNS is illuminated by a normally incident *x*-polarized light. The spectra as well as the electromagnetic field distributions of the PNS presented in this paper are calculated by using the finite element method commercial software of COMSOL Multiphysics. As shown in Fig. 1c, there is no Fano resonance for the non-shrunk PNS with Δ = 0. However, two Fano resonances with 100% modulation depths (defined as the transmission differences between Fano peaks and Fano dips) can be obtained by slightly shrinking or expanding the nanoholes. Comparing with the transmission response of the non-shrunk PNS, the transmission response of the shrunk PNS varies abruptly while the sidebands are kept almost the same.

**Fig. 2:**
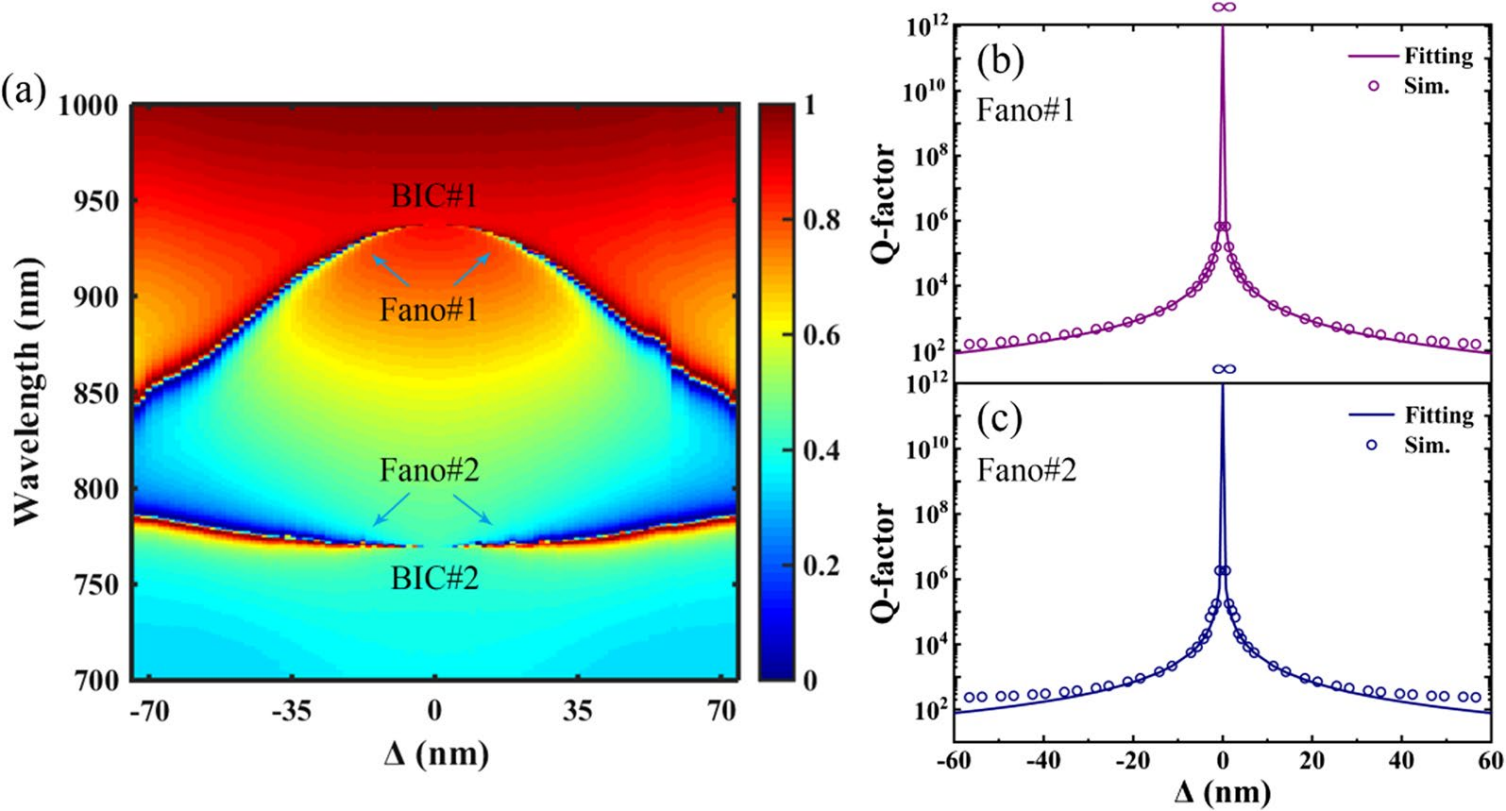
**a** Transmission 2D map of the PNS as a function of the shift distance of Δ along the diagonals of the superlattice. **b** and **c** Q-factor and the fitting result of Fano#1 and Fano#2, respectively. Other parameters are the same as Fig. 1c

To clearly show the evolution of the dual-band Fano resonances arising from the shrinking or expanding of the tetramerized holes, transmission 2D map of the PNS as a function of the shift distance of Δ is shown in Fig. 2a. As shown in Fig. 2a, two BICs are occurred in the wavelength region of interest as Δ = 0, and similar phenomenon of dual BICs was previously reported in the structures of dual-grating metamembranes [13] and split ring resonator [21]. In the case of Δ ≠ 0, dual-band Fano resonances are realized as BICs are induced to QBICs due to the symmetry breaking of the PNS, i.e., from the centrosymmetry of simple lattice to the fourfold rotational (C_4_) symmetry of superlattice. In addition, because the C_4_ symmetry of the PNS can be maintained as the tetramerized holes are shrunk or expanded along the diagonals of the superlattice, the transmission spectra of the PNS are the same for the same absolute value of |Δ|. In principle, the shrink or expansion of the tetramerized holes reduces the area of the first Brillouin zone of the PNS as the unit cell of the PNS changes from simple lattice to superlattice, and symmetry-protected BIC can be excited at normal incidence due to the introduction of surface perturbation as well as Brillouin zone folding of the PNS [42, 43]. Generally, the Q-factor of a symmetry-protected BIC shows an inverse square dependence on the degree of asymmetry *δ* based on the perturbation theory [21]:

1 $$Q_{fit} = \kappa \cdot \frac{cS}{{\omega \cdot \delta^{2} }},$$

where *ĸ* is a proportionality constant, *S* is the area of a superlattice, *ω* is the angular frequency and the asymmetry parameter is $\delta { = }\sqrt 2 \Delta /\Lambda$.

Figure 2b, c shows the Q-factor and the fitting result of Fano#1 and Fano#2, respectively. The Q-factor of the PNS is calculated as a ratio between the resonance wavelength *λ*_*r*_ and its full width at half maximum (FWHM) Δλ, where Δλ is the wavelength region between the peak and dip of Fano resonance. The fitting results of the PNS are calculated by using Eq. (1). As shown in Fig. 2b, c, diverging trajectories of the PNS where the Q-factors diverge to infinity at Δ = 0 are validated by using the inverse square relation to fit the data. Excellent fitting results can be obtained and the slight disagreement at larger asymmetry is due to the deviation from the assumption of tiny perturbation in Eq. (1). The significant advantage of the PNS is that the location and the Q-factor of the dual-band Fano resonances can be tailored by shrinking or expanding the tetramerized holes, which facilitates the dynamic control of the resonant performances of the high-Q-factor multiple Fano resonances.

### Physical mechanisms and interpretation

To get insight into the origin of the dual-band Fano resonances via shrinking or expanding the tetramerized holes of the PNS, we decompose the far-field radiation of BIC and Fano resonance into contributions of different multipole components to further discuss their features. The multipole moments can be calculated based on the displacement current density ***j*** in the superlattice of the PNS [26, 44, 45]:

2 $${\varvec{P}} = \frac{1}{i\omega }\int {{\varvec{j}}d^{3} r} ,$$

3 $${\varvec{M}} = \frac{1}{2c}\int {\left( {{\varvec{r}} \times {\varvec{j}}} \right)d^{3} r} ,$$

4 $${\varvec{T}} = \frac{1}{10c}\int {\left[ {\left( {{\varvec{r}} \cdot {\varvec{j}}} \right){\varvec{r}} - 2r^{2} {\varvec{j}}} \right]} d^{3} r,$$

5 $${\varvec{Q}}_{\alpha ,\beta }^{\left( e \right)} = \frac{1}{i2\omega }\int {\left[ {r_{\alpha } j_{\beta } + r_{\beta } j_{\alpha } - \frac{2}{3}\left( {{\varvec{r}} \cdot {\varvec{j}}} \right)}\delta _{\alpha ,\beta }\right]} d^{3} r$$

6 $${\varvec{Q}}_{\alpha ,\beta }^{\left( m \right)} = \frac{1}{3c}\int {\left[ {\left( {{\varvec{r}} \times {\varvec{j}}} \right)_{\alpha } r_{\beta } + \left( {{\varvec{r}} \times {\varvec{j}}} \right)_{\beta } r_{\alpha } } \right]d^{3} r} ,$$

where ***P***, ***M***, ***T***, ***Q***^(*e*)^ and ***Q***^(*m*)^ are the moments of electric dipole (ED), magnetic dipole (MD), toroidal dipole (TD), electric quadrupole (EQ) and magnetic quadrupole (MQ), respectively; *c* is the speed of light in vacuum, and *α*, *β* = *x*, *y*, *z*. Here the charge density *ρ*, which usually appears in the definition of ED and MQ, has been replaced with displacement current density ***j*** via charge conservation relationship of $i\omega \rho + \nabla \cdot {\varvec{j}} = 0$. In the case of harmonic excitation ~ exp(*iωt*), the scattering power of the induced multipole moments contributing to the far-field response can be written as:

7 $$\begin{aligned} I &= \frac{{2\omega^{4} }}{{3c^{3} }}\left| {\varvec{P}} \right|^{2} + \frac{{2\omega^{4} }}{{3c^{3} }}\left| {\varvec{M}} \right|^{2} + \frac{{2\omega^{6} }}{{3c^{5} }}\left| {\varvec{T}} \right|^{2} + \frac{{\omega^{6} }}{{5c^{5} }}\sum\limits_{\alpha ,\beta } {\left| {{\varvec{Q}}_{\alpha ,\beta }^{\left( e \right)} } \right|}^{2} \\ &\quad+ \frac{{\omega^{6} }}{{20c^{5} }}\sum\limits_{\alpha ,\beta } {\left| {{\varvec{Q}}_{\alpha ,\beta }^{\left( m \right)} } \right|}^{2} + {\text{o}}(\omega), \end{aligned}$$

where the first two terms correspond to the conventional ED (charge) and MD scattering. The third term corresponds to the TD scattering. The fourth and fifth terms come from EQ and MQ. The last term is the higher-order term that contains the high-order multipole scattering and coupling between them and can be generally ignored. By using Eqs. (2)–(7), the contributions of different multipoles to the scattering power of the far field can be obtained.

**Fig. 3:**
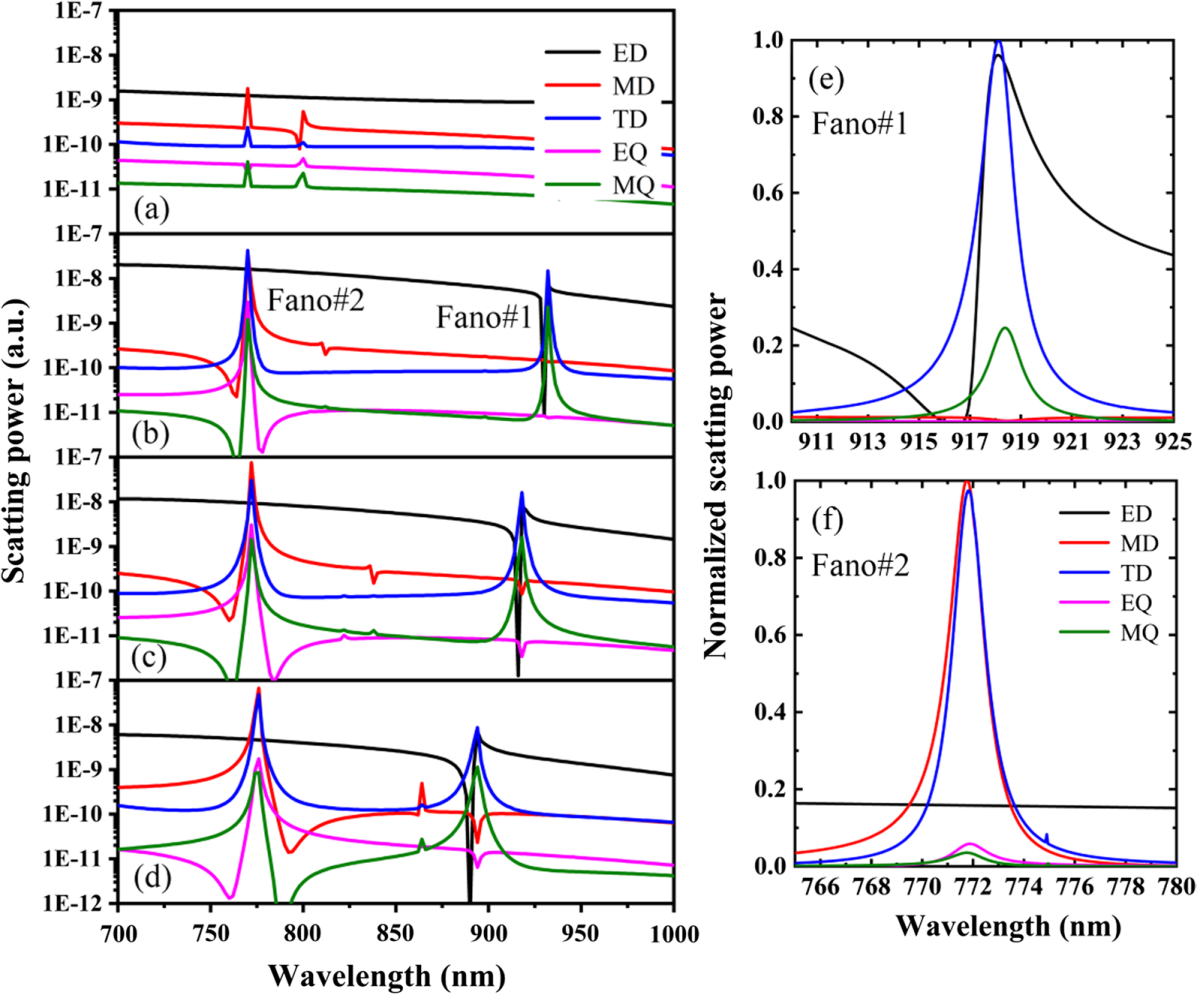
Scattering power of the Cartesian ED, MD, TD, EQ and MQ when **a** Δ = 0, **b** Δ =  − 14 nm, **c** Δ =  − 28 nm and **d** Δ =  − 42 nm. **e** and **f** Normalized scattering power of different multipoles with Δ =  − 28 nm around Fano#1 and Fano#2, respectively. Other parameters are the same as Fig. 1c

Figure 3 shows the scattering power of different multipoles of the PNS for different shift distance of Δ, other parameters are the same as Fig. 1c. As shown in Fig. 3a–d, for the PNS with Δ = 0, ED and MD are the dominate dipoles and they are not resonant at the wavelength region of interest. However, by shrinking or expanding the nanoholes of the PNS with |Δ|≠ 0, dual-band Fano resonances can be realized due to the excitations of the resonant dipole modes. To clearly see the important roles of the resonant dipole modes in forming the observed dual-band Fano resonances, Fig. 3e, f shows the normalized scattering power of different multipoles with Δ =  − 28 nm around Fano#1 and Fano#2, respectively. As shown in Fig. 3e, the dominant resonant modes are ED and TD around Fano#1, and Fano#1 is the direct consequence of the resonant coupling of the electric toroidal dipoles. In particular, ED and TD are strongly enhanced to a comparable magnitude at the resonant tip (918.5 nm) of Fano#1; thus, 100% transmission can be obtained due to the destructive interference between ED and TD. While for the resonant dip (916.5 nm) of Fano#1, the reflection is maximized and the transmission goes to zero due to the enhanced scattering of ED and TD. Similarly, as shown in Fig. 3f, Fano#2 is arising from the resonant coupling of the magnetic toroidal dipoles, its tip (771.1 nm) indicates the destructive interference between MD and TD, while its dip (772.9 nm) is associated with the enhanced scattering of MD and TD. Note due to the strong coupling of electric toroidal dipoles or magnetic toroidal dipoles, the resonant modes are robust for both Fano#1 and Fano#2 even if Δ is varied.

**Fig. 4:**
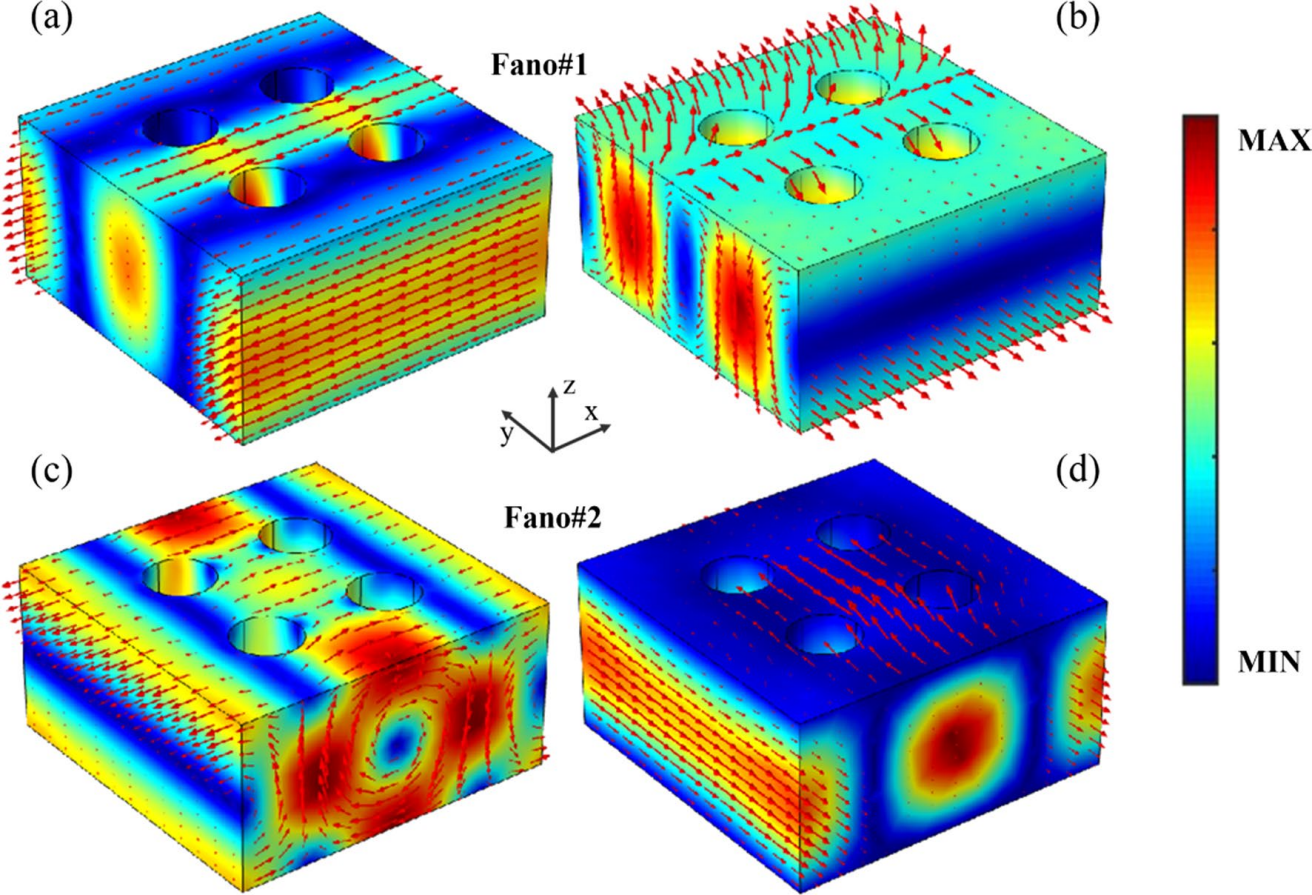
Distributions of electromagnetic field and displacement current of Fano resonances of the superlattice of the PNS, the color bar represents the field amplitude, and the red arrows indicate field vector or displacement current vector. Other parameters are the same as Fig. 1c with Δ =  − 28 nm. **a** and **c** Distributions of electric field amplitude and displacement current vector of Fano#1 and Fano#2, respectively. **b** and** d** Distributions of magnetic field amplitude and magnetic field vector of Fano#1 and Fano#2, respectively

To link the transmission response of dual-band Fano resonances in the far field with the excitations of induced multipole moments, the distributions of electromagnetic field and displacement current of Fano resonances of the superlattice of the PNS are shown in Fig. 4. As shown in Fig. 4a, b, the electric field of Fano#1 is well confined in the superlattice of the PNS with the displacement current along the *x* axis, indicating an ED resonant mode. Moreover, the displacement current of Fano#1 forms two reversed loops between the center and the edges of the superlattice, and the magnetic field forms a loop in the *yz* plane, corresponding to a TD resonance mode along the *x* axis [44, 46]. Therefore, Fano#1 is arisen from the resonant coupling between the ED and TD modes, which are in line with the predicted results of the multipole decompositions as mentioned above. In fact, due to the resonant features of the electric toroidal dipoles of Fano#1, the distributions of electromagnetic field and displacement current at the resonant peak (918.5 nm), central wavelength (917.5 nm) and resonant dip (916.5 nm) of Fano#1 are almost the same, except a slight difference in the field amplitude (Additional file 1: Fig. S1). In the case of Fano#2, as shown in Fig. 4c, the electric field is strongly enhanced and the displacement current forms two reversed loops between the center of the superlattice and the neighboring superlattice of the PNS, indicating a TD resonance mode along the *z* axis. Besides, the magnetic field of Fano#2 is highly localized in the superlattice with the direction along the *y* axis, as shown in Fig. 4d, featuring a MD resonant mode. As a result, Fano#2 is the direct consequence of the resonant coupling the magnetic toroidal dipoles, which is in agreement with the prediction of the multipole decompositions of the far field of the PNS. Also, due to the coupling of the magnetic toroidal dipoles of Fano#2, the electromagnetic field and displacement current at the resonant peak (771.1 nm), central wavelength (772.0 nm) and resonant dip (722.9 nm) of Fano#2 show similar distributions (Additional file 1: Figure S2).

## Results and discussion

**Fig. 5:**
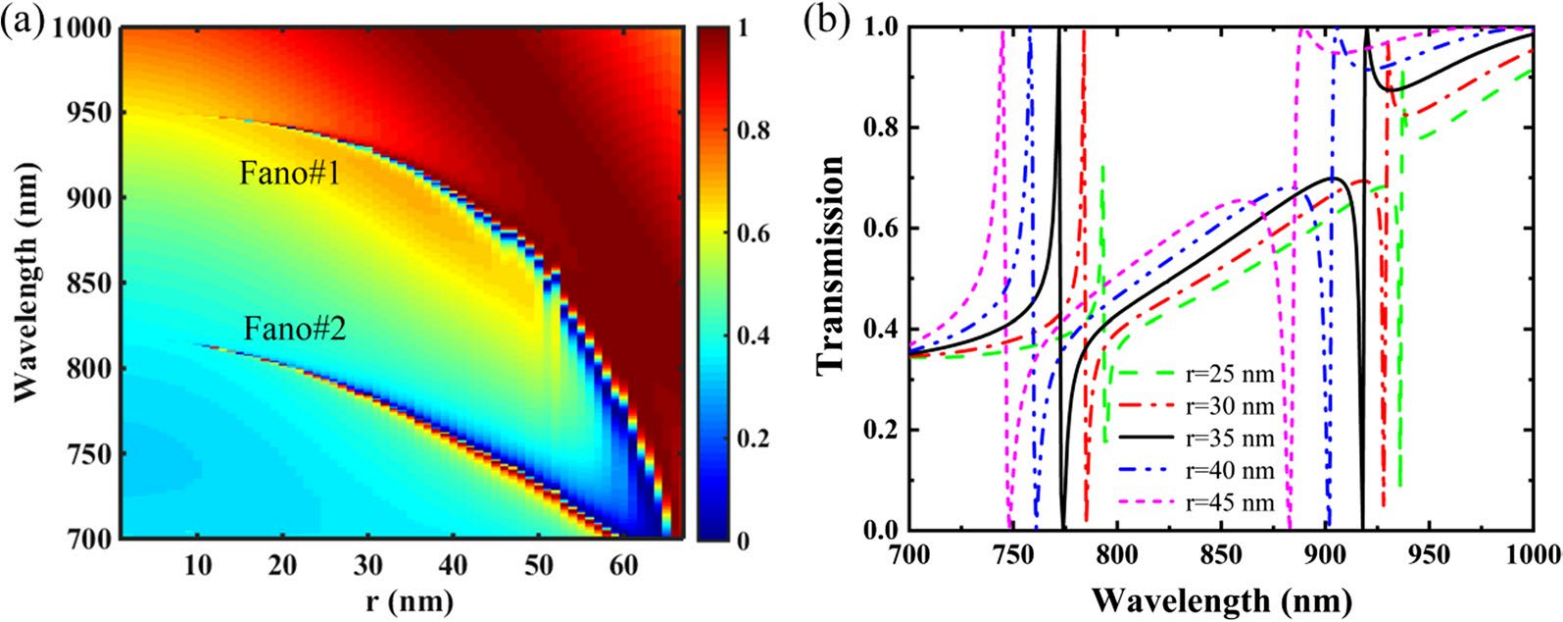
**a** Transmission 2D map of the PNS as a function of the radius *r* of the nanohole. **b** Transmission spectra of the PNS for different nanohole radius *r*. Other parameters are the same as Fig. 1c with Δ =  − 28 nm

Figure 5 shows transmission spectra of the PNS as a function of the radius *r* of the nanohole, and other parameters are the same as Fig. 1c with Δ =  − 28 nm. As shown in Fig. 5a, dual-band Fano resonances can be maintained as *r* is varied from 0 to the maximum value of 67.5 nm, *i.e.*, the tetramerized holes are tangent to each other in the superlattice. The increase in the nanohole radius *r* increases the surface perturbations of the PNS and reduces its effective refractive index (ERI) as well, resulting in the increased Q-factor and the blueshift of the Fano resonances. Specifically, the resonant location of Fano#1 is more sensitive to the variation of *r*, and the dual-band Fano resonances tend to merge into one resonant mode as the tetramerized holes approach each other. As shown in Fig. 5b, the increase of *r* not only blueshifts the resonant location of the Fano resonances but also increases their FWHMs. As *r* is increased from 25 to 45 nm, the resonant peaks of Fano#1 and Fano#2 are blueshifted from 936.7 nm and 793.2 nm to 887.6 nm and 743.8 nm, respectively; and their FWHMs are increased from 0.8 nm and 0.6 nm to 6.8 nm and 3.1 nm, respectively. Note the increase of *r* also improves the modulation depths of the Fano resonances, and 100% modulation depths can be realized as *r* is larger than 30 nm. Additionally, by evaluating the shift of Fano peak wavelength affected by the structural parameters of the PNS, it is shown that the nanohole radius *r* is the most sensitive structural parameters for both Fano#1 and Fano#2 (Additional file 1: Figure S3). Therefore, the variation of *r* provides an effective approach to dynamically control the resonant performances of the dual-band Fano resonances of the PNS.

**Fig. 6:**
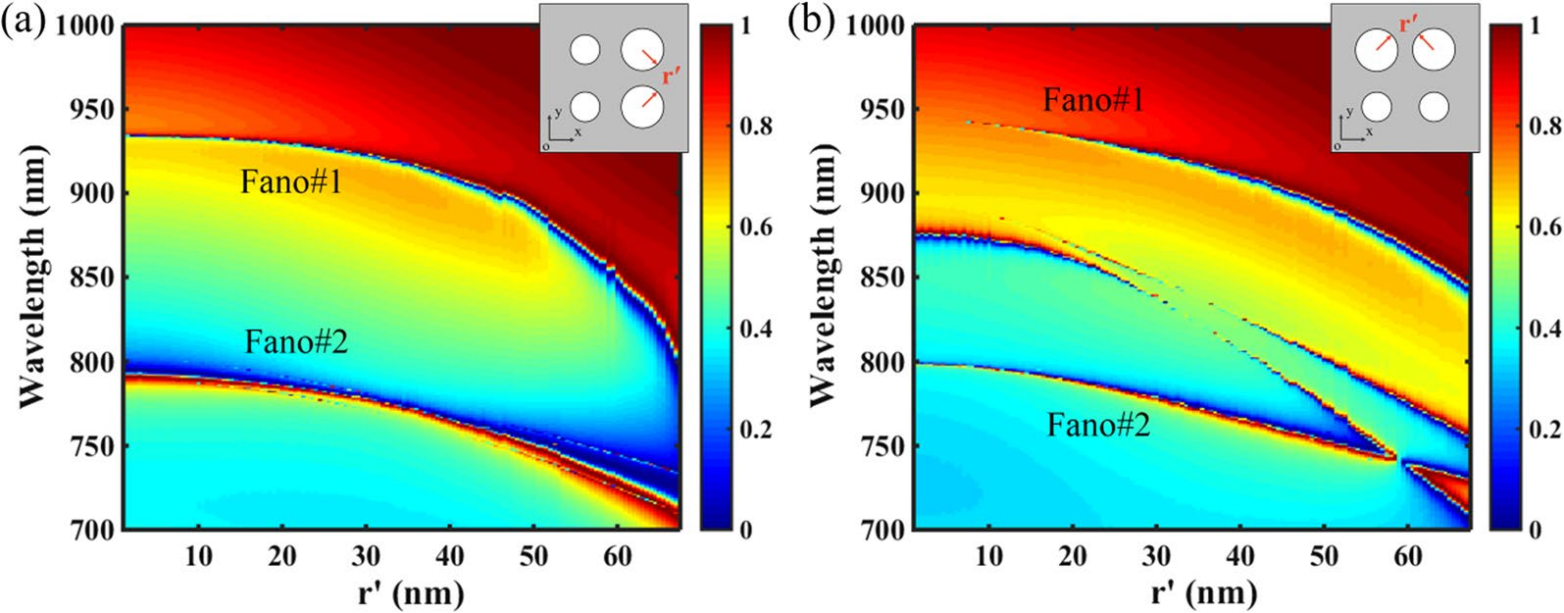
Influences of the symmetry of the structure on transmission responses of the PNS. Other parameters are the same as Fig. 1c with Δ =  − 28 nm. The insert figures indicate the schematic diagram of the superlattice of the PNS. **a** Transmission 2D map of the PNS as a function of the radius *r*′ of two nanoholes, where the structural symmetry of the superlattice along the *x* axis is maintained. **b** Transmission 2D map of the PNS as a function of the radius *r*′ of two nanoholes, where the structural symmetry of the superlattice along the *x* axis is broken

Figure 6 shows the influences of the symmetry of the structure on transmission responses of the PNS, where the radius *r'* of two nanoholes is varied from zero to tangent to each other, and other parameters are the same as Fig. 1c with Δ =  − 28 nm. As shown in Fig. 6a, for the superlattice with mirror symmetry along the *x* axis (direction of electric field of incident light), as the radius *r*′ of the two nanoholes is increased, the resonant locations of the dual-band Fano resonances are blueshifted due to the decrease in the ERI of the PNS, and their bandwidths are broaden due to the increased surface perturbations. However, as shown in Fig. 6b, although the two Fano resonances can be maintained with the increase of *r*′, two additional Fano resonances will occur as the mirror symmetry of the superlattice along the *x* axis is broken. In general, breaking the structural symmetry along the *x* (*y*) axis will also break the symmetry of the mode along the *x* (*y*) axis of a periodic lattice, and the non-radiative non-degenerate mode is able to couple to outside radiation due to its degenerate component [47]. Therefore, the fact that the two additional Fano resonances are present for only the mirror symmetry-broken structure along *x* axis indicates that they are due to the perturbed non-degenerate modes.

**Fig. 7:**
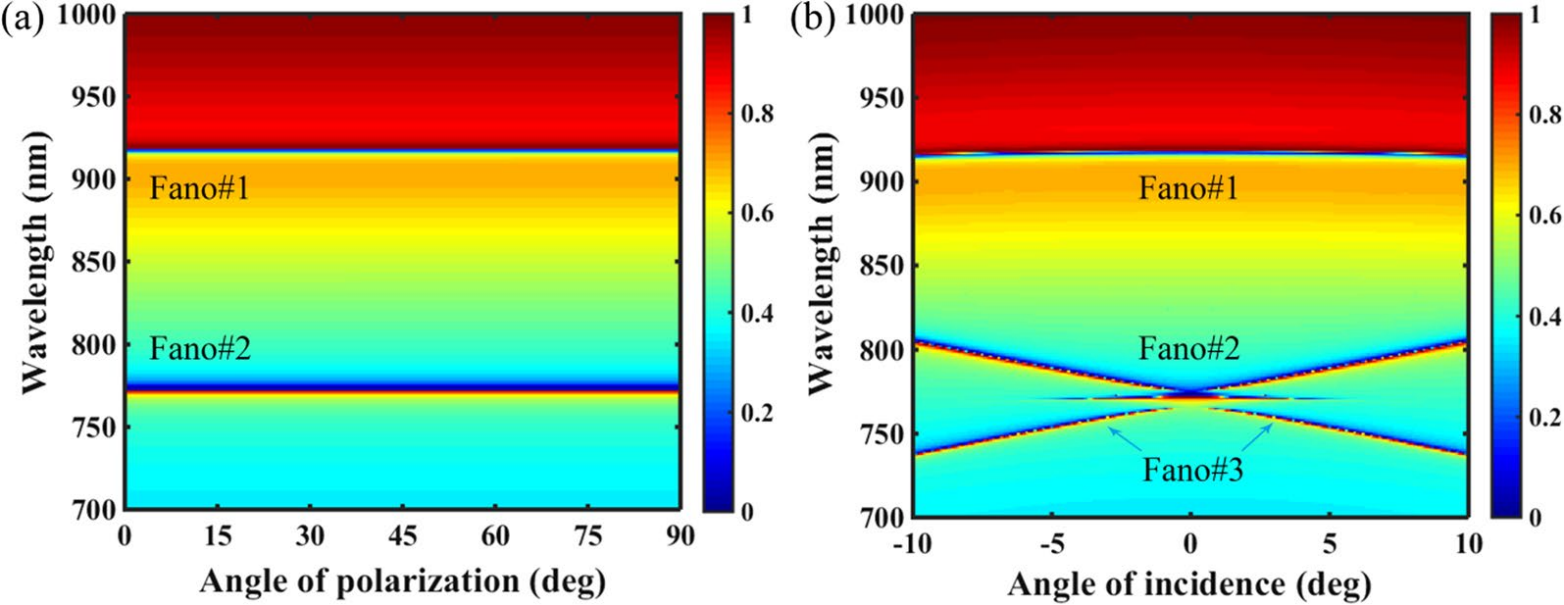
**a** Transmission 2D map of the PNS as a function of the polarization angle. **b** Transmission 2D map of the PNS as a function of the incident angle. Other parameters are the same as Fig. 1c with Δ =  − 28 nm

We further characterized the resonant performances of the PNS under the influences of incident angle and polarization angle. As shown in Fig. 7a, dual-band Fano resonances of the PNS are immune to the variation of polarization angle due to the C_4_ symmetrical topology. As the polarization angle is altered from 0 to 90°, that is, from *x*-polarization to *y*-polarization, Fano#1 and Fano#2 are kept the same. However, in the case of incident angle, as shown in Fig. 7b, although Fano#1 is also insensitive to the variation of the incident angle, Fano#2 is redshifted as the incident angle deviates from normal incidence, and an additional Fano resonance (Fano#3) is occurred due to the radiation decay suppression of the symmetry-protected BIC is canceled at off-normal incidence. In general, this type of radiation decay suppression of BIC is closely related to the destructive interference between the emitted radiation fields from two counter-propagating leaky modes at either one of two edges of the stop band of the periodic lattices [48]. Note due to the strong coupling between Fano#2 and Fano#3, a narrow induced transparency window can be excited in the vicinity region between them.

**Fig. 8:**
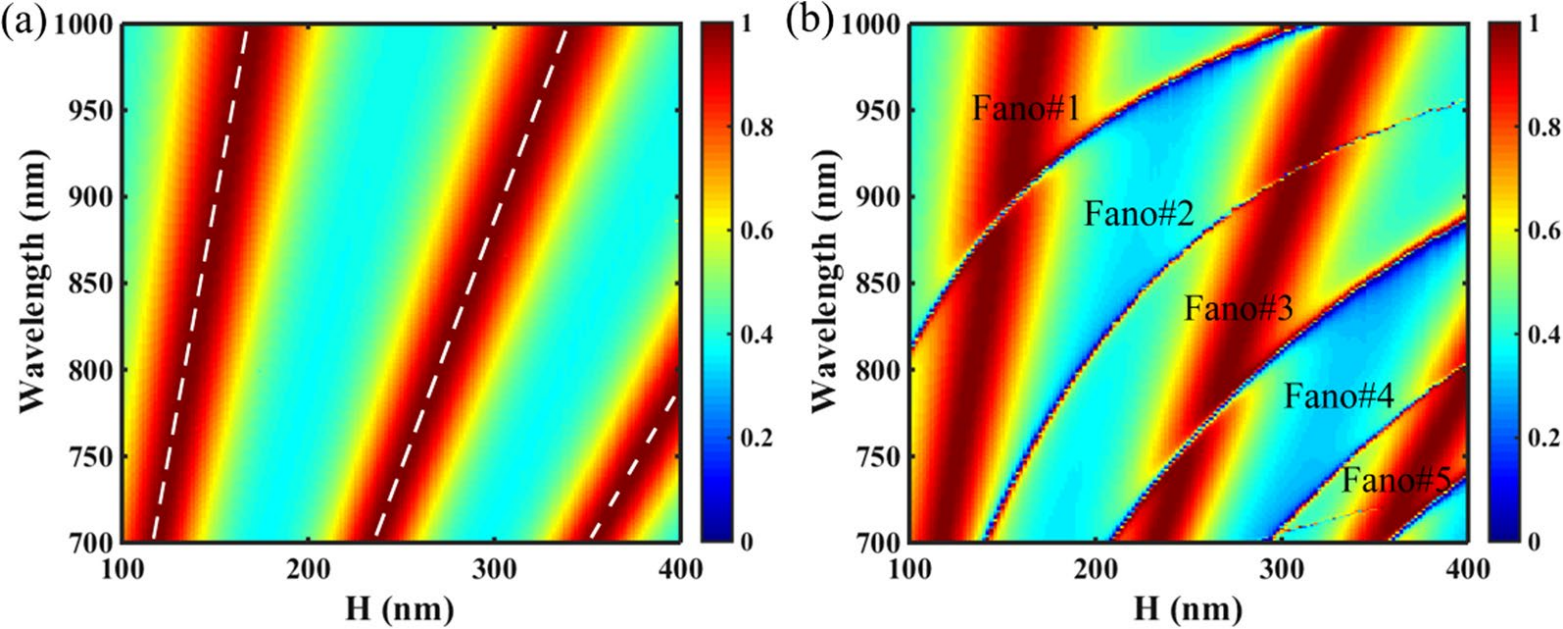
**a** Transmission 2D map of the PNS as a function of the slab height H with Δ = 0 nm, the white dash lines are the results of the F–P cavity model. **b** Transmission 2D map of the PNS as a function of the slab height H with Δ =  − 28 nm. Other parameters are the same as Fig. 1c

Finally, we showed that multiple Fano resonances can be obtained by increasing the slab height H of the PNS. Figure 8 shows the transmission 2D map of the PNS as functions of H for the non-shrunk (Δ = 0 nm) and shrunk (Δ =  − 28 nm) structures. As shown in Fig. 8a, there is no Fano resonance except the Fabry–Pérot (F–P) resonances for the non-shrunk PNS as H is varied. According to the F–P theory, the resonance condition of the F–P cavity of the non-shrunk PNS can be written as:

8 $$\delta = (2\pi /\lambda ) \cdot H \cdot n_{eff} + \varphi = m\pi ,$$

where *δ* is the phase shift, *λ* is the wavelength in free space, *n*_*eff*_ is the ERI of the equivalent homogeneous slab of the PNS, *φ* is the additional phase and *m* is an integer which indicates the resonance order. By using the effective medium theory [49], the ERI of the PNS can be estimated as:

9 $$n_{eff} = \sqrt {\frac{{\left[ {\left( {1 - f} \right)n_{a}^{2} + fn_{s}^{2} } \right]\left[ {fn_{a}^{2} + \left( {1 - f} \right)n_{s}^{2} } \right] + n_{s}^{2} }}{{2\left[ {fn_{a}^{2} + \left( {1 - f} \right)n_{s}^{2} } \right]}}} ,$$

where *f* is the filling factor of the PNS, and *f* = 1 − 4π(*r*/Λ)^2^.

By using Eqs. (8) and (9), the locations of the F–P resonance of the non-shrunk PNS can be calculated as *λ*_*F–P*_ = 2*π*·H·*n*_*eff*_/(*mπ*-*φ*). In calculation, although the additional phase *φ* cannot be treated as a constant because it evidently affects phase shift *δ*, its values can be figured out by using the linear fitting method [50, 51]. Figure 8a shows the transmission 2D map of the PNS with Δ = 0 nm, and the results of the F–P theory are indicated by the white dash lines. As shown in Fig. 8a, the white dash lines of the F–P cavity model are coincided with those of the transmission peaks of the PNS, confirming it is the F–P resonance that enhances the transmission of the non-shrunk PNS in the spectral region of interest. However, as shown in Fig. 8b, for the shrunk PNS with Δ =  − 24 nm, five Fano resonances with high Q-factor are excited and coexisted with the F–P resonances as H is varied in the range of 100–400 nm, the Fano resonances are so strong that they split the F–P resonances in the crossing region between the Fano and F–P resonances. According to the slab waveguide theory, the increase in the thickness of the photonic crystal slab ensures more leaky modes bounded in the structure [32, 52]; thus, the number of the Fano resonances can be increased by merely increasing the thickness of the PNS. Note the shift of the tetramerized holes will not change the ERI of the PNS, thus the locations of the F–P resonances are kept almost the same for both the non-shrunk and shrunk structures.

## Conclusions

High-Q-factor dual-band Fano resonances can be realized by using a comparatively simple architecture of PNS based on the excitation of dual QBICs. By shrinking or expanding four nanoholes of the PNS along the diagonals of the superlattice, two symmetry-protected BICs can be transformed to dual-band Fano resonances and their locations as well as their Q-factors can be flexibly tuned. The dual-band Fano resonances of the PNS are resulted from the resonant couplings between the electric toroidal dipoles or the magnetic toroidal dipoles, and their correlations between the far-field multiple decompositions and the near-field distributions of the superlattice are verified. The dual-band Fano resonances of the PNS possess polarization-independent feature, and their high-Q-factor features are robust to the variations of the geometric parameters. By increasing the height of the PNS, the number of high-Q-factor Fano resonances can be improved as more leaky modes can be supported by the structure. Our results give more tuning freedoms for the realization of high-Q-factor resonators with better performances, which may provide a further step in the development of lasing, sensing and nonlinear photonics.

## Supplementary Information

**Additional file 1.Figure S1.** Distributions of electromagnetic field and displacement current of Fano#1. **Figure S2.** Distributions ofelectromagnetic field and displacement current of Fano#2. **Figure S3.** Peak wavelengths of Fano#1 and Fano#2 as functions structural parameters of the PNS.

## Data Availability

The datasets used and/or analyzed during the current study are available from the corresponding author on reasonable request.

## Abbreviations

Q-factor: Quality factor
PNS: Planar nanohole slab
BICs: Bound states in the continuum
NIR: Near-infrared
QBICs: Quasi-BICs
FWHM: Full width at half maximum
ED: Electric dipole
MD: Magnetic dipole
TD: Toroidal dipole
EQ: Electric quadrupole
MQ: Magnetic quadrupole
ERI: Effective refractive index
F–P: Fabry–Pérot
