# Supplementary material for: High-quality-factor dual-band Fano resonances induced by dual bound states in the continuum using a planar nanohole slab
Source: Nanoscale Res Lett. 2021 Sep 28;16:150. doi: 10.1186/s11671-021-03607-x (PMC8479049; doi:10.1186/s11671-021-03607-x)
Supplement: Supplementary file 1 — Additional file 1.Figure S1. Distributions of electromagnetic field and displacement current of Fano#1. Figure S2. Distributions ofelectromagnetic field and displacement current of Fano#2. Figure S3. Peak wavelengths of Fano#1 and Fano#2 as functions structural parameters of the PNS. [file 11671_2021_3607_MOESM1_ESM.docx]

High quality-factor dual-band Fano resonances induced by dual bound states in the continuum using a planar nanohole slab

Qing Mi^1^, Tian Sang^1*^, Yao Pei^1^, Chaoyu Yang^1^, Shi Li^1^, Yueke Wang^1^ and Bin Ma^2^

^1^ Department of Photoelectric Information Science and Engineering, School of Science, Jiangnan University, Wuxi 214122, China

^2^ Key Laboratory of Advanced Micro-Structured Materials MOE, Institute of Precision Optical Engineering, School of Physics Science and Engineering, Tongji University, Shanghai 200092, China

^*^Correspondence: sangt@jiangnan.edu.cn

To better show the resonant properties of the dual-band Fano resonances of the PNS, distributions of electromagnetic field and displacement current at resonant peak, central wavelength and resonant dip of Fano#1 and Fano#2 are demonstrated in Fig. S1 and Fig. S2, respectively. As shown in Fig. S1, the distributions of electromagnetic field and displacement current at the resonant peak (918.5 nm), central wavelength (917.5 nm) and resonant dip (916.5 nm) of Fano#1 are almost the same due to the resonant features of the electric-toroidal dipoles. As can be seen in Fig. S2, in the case of Fano#2, the electromagnetic field and displacement current at the resonant peak (771.1 nm), central wavelength (772.0 nm) and resonant dip (722.9 nm) also show similar distributions due to the resonant magnetic-toroidal dipoles.


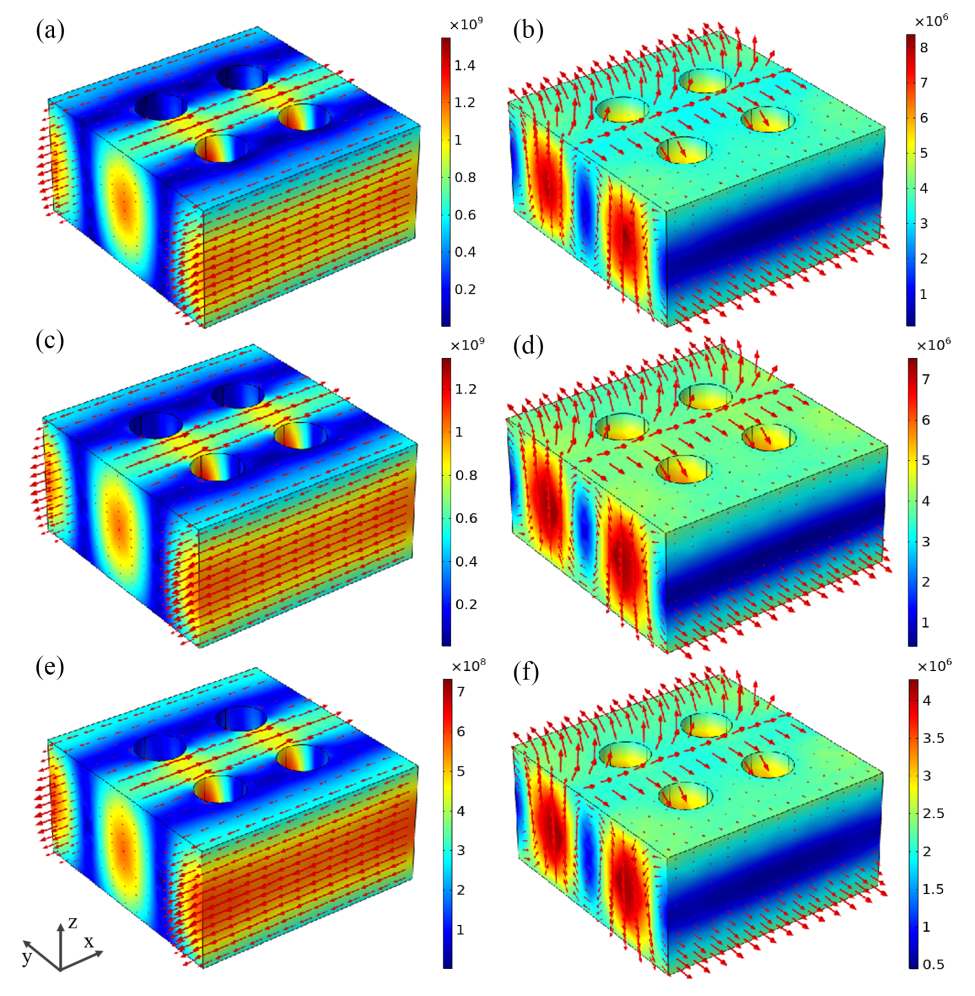


**Figure S1.** Distributions of electromagnetic field and displacement current of Fano#1, the color bar represents the field amplitude, other parameters are the same as Fig. 1c with Δ=-28 nm. (a), (c) and (e) are distributions of electric field amplitude and displacement current vector of resonant peak (918.5 nm), central wavelength (917.5 nm) and resonant dip (916.5 nm), respectively. (b), (d) and (f) are the distributions of magnetic field amplitude and magnetic field vector of resonant peak (918.5 nm), central wavelength (917.5 nm) and resonant dip (916.5 nm), respectively.


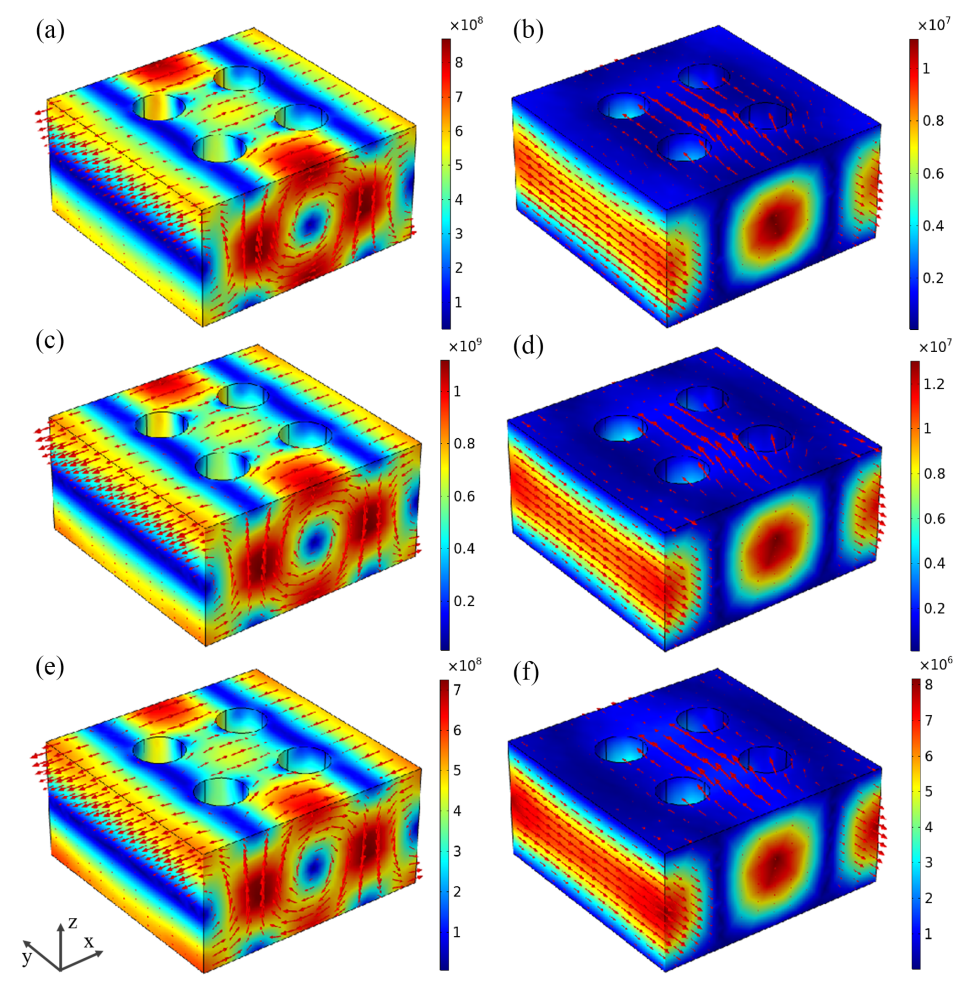


**Figure S2.** Distributions of electromagnetic field and displacement current of Fano#2, the color bar represents the field amplitude, other parameters are the same as Fig. 1c with Δ=-28 nm. (a), (c) and (e) are distributions of electric field amplitude and displacement current vector of resonant peak (771.1 nm), central wavelength (772.0 nm) and resonant dip (722.9 nm), respectively. (b), (d) and (f) are distributions of magnetic field amplitude and magnetic field vector of resonant peak (771.1 nm), central wavelength (772.0 nm) and resonant dip (722.9 nm), respectively.

Figure S3 show sensitivity of Fano peak wavelength affected by the structural parameters of the PNS, and the slope of the curve in the figure indicates the sensitivity. As can be seen in Fig. S3(a), due to the complex coupling of the electromagnetic field confined in the tetramerized holes, the increase of Δ results in obvious blue-shift (slope<0) of the peak wavelength of Fano#1, while the peak wavelength of Fano#2 is slightly red-shifted (slope>0). In Figs. S3(b)-(d), because the increase of the nanohole radius decreases the ERI of the PNS, the peak wavelengths for both Fano#1 and Fano#2 are blue-shifted as the nanohole radius is increased. However, as shown in Fig. S3(e), the increase of slab height H increases the optical thickness of the PNS, thus the peak wavelength are all red-shifted for both Fano#1 and Fano#2. By comparing the slope of the curves for different structural parameters, it can be seen that the nanohole radius *r* is the most sensitive structural parameters for both Fano#1 and Fano#2. That is, the increase of 1 nm *r* results in the peak wavelength shift of 1.91 nm and 2.08 nm for Fano#1 and Fano#2, respectively. While the peak wavelengths of Fano#1 and Fano#2 are less sensitive to the variations of the slab thickness H and the shift distance of Δ, respectively. The increase of 1 nm H shifts the peak wavelength of Fano#1 0.86 nm, and the increase of 1 nm Δ shifts the peak wavelength of Fano#2 0.20 nm.


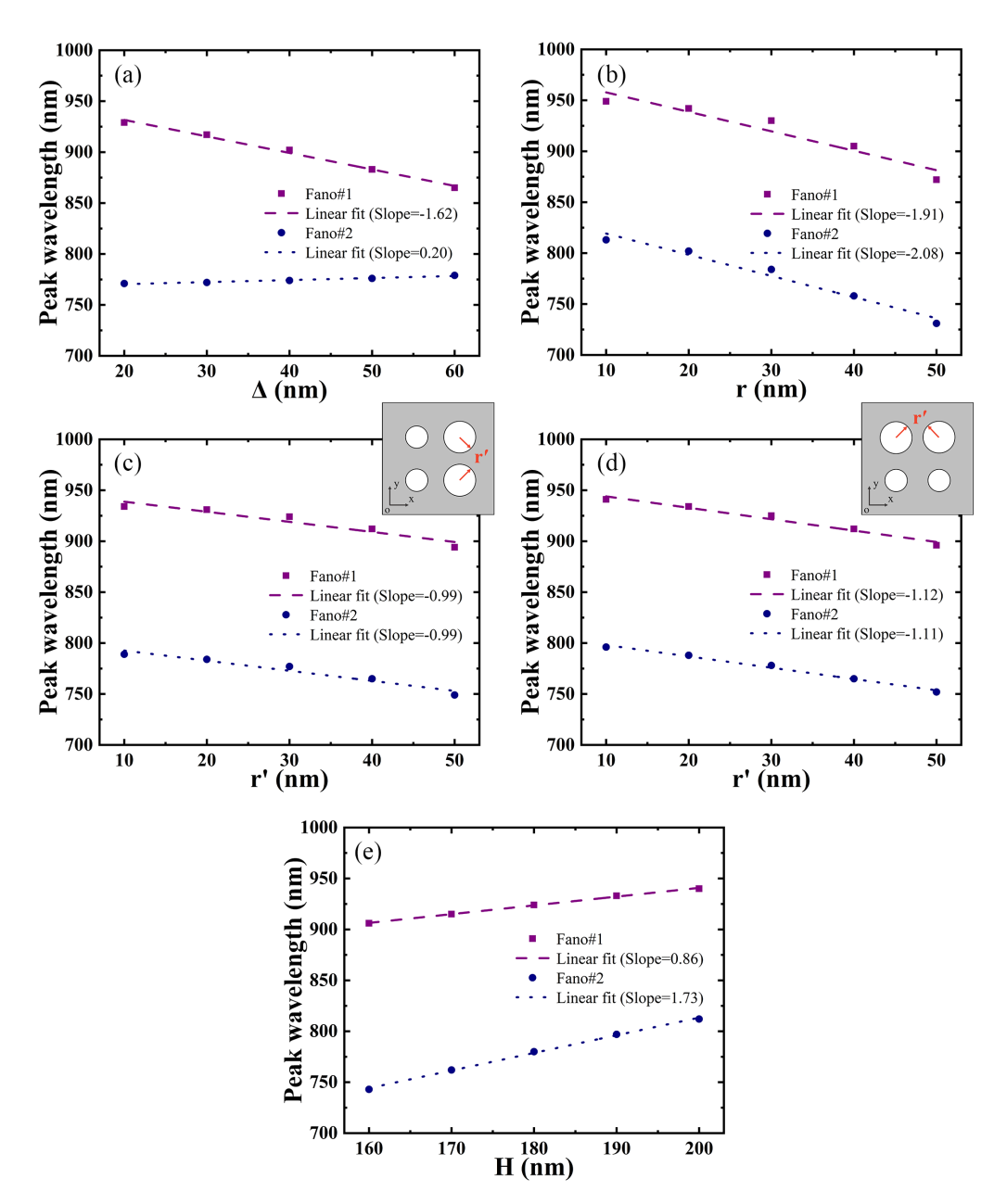


**Figure S3.** Peak wavelengths of Fano#1 and Fano#2 as functions of (a) shift distance Δ, (b) nanohole radius *r*, (c) right nanohole radius *r'*, (d) top nanohole radius *r'*, and (e) slab height H of the PNS. Other parameters are the same as Fig. 1c.
